# The Effects of High Fat Diet-Induced Stress on Olfactory Sensitivity, Behaviors, and Transcriptional Profiling in *Drosophila melanogaster*

**DOI:** 10.3390/ijms19102855

**Published:** 2018-09-20

**Authors:** Jewon Jung, Dong-In Kim, Gi-Youn Han, Hyung Wook Kwon

**Affiliations:** Department of Life Sciences and Convergence Research Center for Insect Vectors, Incheon National University, 119 Academy-ro, Yeonsu-gu, Incheon 22012, Korea; jjewony@gmail.com (J.J.); kdin34@naver.com (D.-I.K.); bkdgy93@gmail.com (G.-Y.H.)

**Keywords:** *Drosophila melanogaster*, high fat diet, olfaction, olfactory receptors, gene expression, climbing behaviors

## Abstract

High-fat diet (HFD) often causes obesity and it has detrimental effects on the sensory system. In particular, sensory-mediated responses are crucial for maintaining energy balance, as they are involved in a metabolic regulation; however, there is still no clear explanation about the relationship between HFD-induced stress and sensory system. To gain insight on how HFD-induced stress affects olfactory sensitivity and behavioral responses, we have used a *Drosophila melanogaster* model for olfactory and nutrient-related signaling and accessed physiological, behavioral, and transcriptional changes. We demonstrated that lifespan and climbing ability in HFD-treated flies decreased and that olfactory sensitivity and behavioral responses to odorants were changed. Olfactory sensitivity to eight of ten odorants after 14 days on HFD treatment were reduced, while behavioral attraction was increased to benzaldehyde in flies that were treated with HFD. This behavioral and physiological modification in HFD-treated flies for 14 days was accompanied by a significant decrease in *DmOrco* gene expression in a peripheral olfactory organ, suggesting that is could be involved in the action of metabolic and sensory signal. Gene expression profiles of antennae showed significant differences on the olfactory receptors, odorant-binding proteins, and insulin signaling. Our results suggested that olfactory sensitivity and behavioral responses to HFD-induced stress are mediated through olfactory and nutrient-related signaling pathways.

## 1. Introduction

Most organisms maintain energy homeostasis to survive and function effectively under the influence of internal and external factors for biological processes of growth, development, metabolism, and aging [1,2]. Many environmental conditions, including nutrients, temperature, and light can influence food intake and cause consequences for health, including obesity [3,4]. Obesity causing a myriad of complex diseases is known to be related with an imbalance in the homeostasis of energy metabolism [5,6,7]. A previous study reported that simultaneous processing in taste and olfaction, the pivotal senses for survival, is associated with human obesity [8]. For instance, food intake controlled by external sensory signals has an effect on energy balance assessed by internal metabolic signals [9]. These fundamental responses are made through physiological changes, metabolic hormones, in particular, which regulate appetite [10].

Sensory-mediated responses are remarkably complex network [11,12]. In the natural environment, olfaction is related to finding food sources, recognizing predators, and locating mates [13,14]. In particular, feeding behaviors are closely linked and initiated with the finding of food sources in response to sensory inputs, for instance, olfactory information [12]. In mammals, such metabolic regulation in the olfactory cortex is changed by internal metabolic states where an increased number of insulin receptors (InR) on the olfactory bulb was found in starved mice [15] suggesting that the regulation of insulin signaling by food intake was attributed to olfaction.

*Drosophila melanogaster*, the fruit fly that is a powerful tool for studying gain- and loss-of-function by observing phenotypic changes and behavioral analysis, possesses evolutionarily conserved pathways in common with mammals [16,17]. Evidence for the link between olfactory response and insulin signaling has been shown by flies fed high-fat diet (HFD) exhibiting decreased olfactory sensitivity and odor-driven food searching behavior in comparison with flies that were fed with normal diet [18]. However, little is known about the impact of food stress on olfactory signaling and nutrient-related genes in the main peripheral olfactory organ, the antennae, of *Drosophila melanogaster*. It has been suggested that preferential odorant of over-weight people appeared to be a food-related rather than a non-food related [8]. Given this evidence, the alteration of metabolism and enhancement of stress resistance by the olfactory system in relation to food-derived odors might be involved in the modulation of the lifespan. Furthermore, these findings are likely to be correlated with feeding behavior and the energy balance through sensory processing [19,20,21].

To gain insight into how HFD-induced stress affects olfactory sensitivity and behaviors that mediate nutrient-related signaling, this study centers on the understanding of behavioral and sensory modification by food stress, such as HFD treatment in *D. melanogaster,* and it employs lifespan analysis, behavioral tests such as a climbing test, a choice test for food-related odorants, and electrophysiological recordings from antennae (Electroantennogram, EAG). Moreover, to pinpoint any modifications at the molecular level in the main olfactory organ, we examined the expression levels of *DmOrco* gene by quantitative real-time PCR (qRT-PCR) and overall transcriptional profiles of antennal tissues to identify genes including olfactory and nutrient-related signaling genes whose levels fluctuated with HFD treatment. These approaches provide a general overview of relationships between food stress and sensory modulation machineries in the peripheral olfactory organ based on Gene Ontology (GO).

## 2. Results

### 2.1. Life Span and Climbing Abilities of Flies Fed with HFD

**Figure 1:**
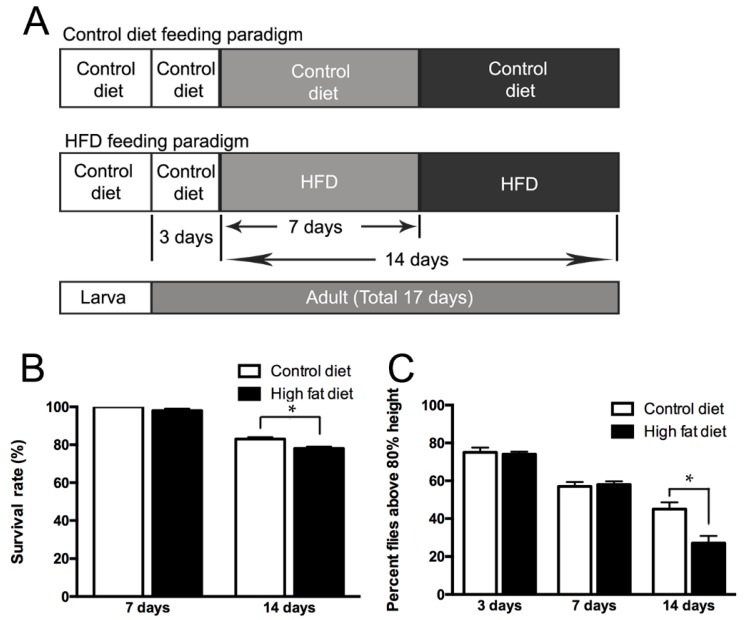
Physiological effects of high-fat diet (HFD) intake on wild type Canton-S flies. (**A**) Food treatment condition and feeding paradigm. The control diet and HFD were treated ad lib for seven and 14 days in male Canton-S flies. (**B**) Lifespan of wild flies fed with control diet and HFD. (*n* = 5, * *p* < 0.05). Error bars represent SEM. (**C**) Locomotor activity of wild type Canton-S flies fed with control diet and HFD. (*n* = 10, * *p* < 0.05). Error bars represent SEM.

We hypothesized that high fat diet (HFD), causing the alteration of physiological states [22,23], might reduce the life span and climbing ability of flies. To confirm the hypothesis, we fed a control diet and high-fat diet (HFD) ad libitum to male Canton-S adult flies four days after eclosion for seven days or 14 days (Figure 1A), with slight modification from the previous study [22]. The results showed that the life span of the flies fed with HFD diet started to decrease at day 7 and it was significantly reduced by approximately 12% at day 14 in comparison with those receiving the control diet (Figure 1B). Consistent with this, there were also indications that the locomotor activity of the flies declined after HFD intake. Since the diet appeared to be associated with climbing ability [24], we measured the climbing ability of flies fed with a control diet or HFD for seven or 14 days. Significant changes in climbing ability were observed for the HFD-treated groups (Figure 1C), suggesting that a HFD has a detrimental influence on fly mobility.

### 2.2. Modification of Olfactory Sensitivity by HFD Treatment

**Figure 2:**
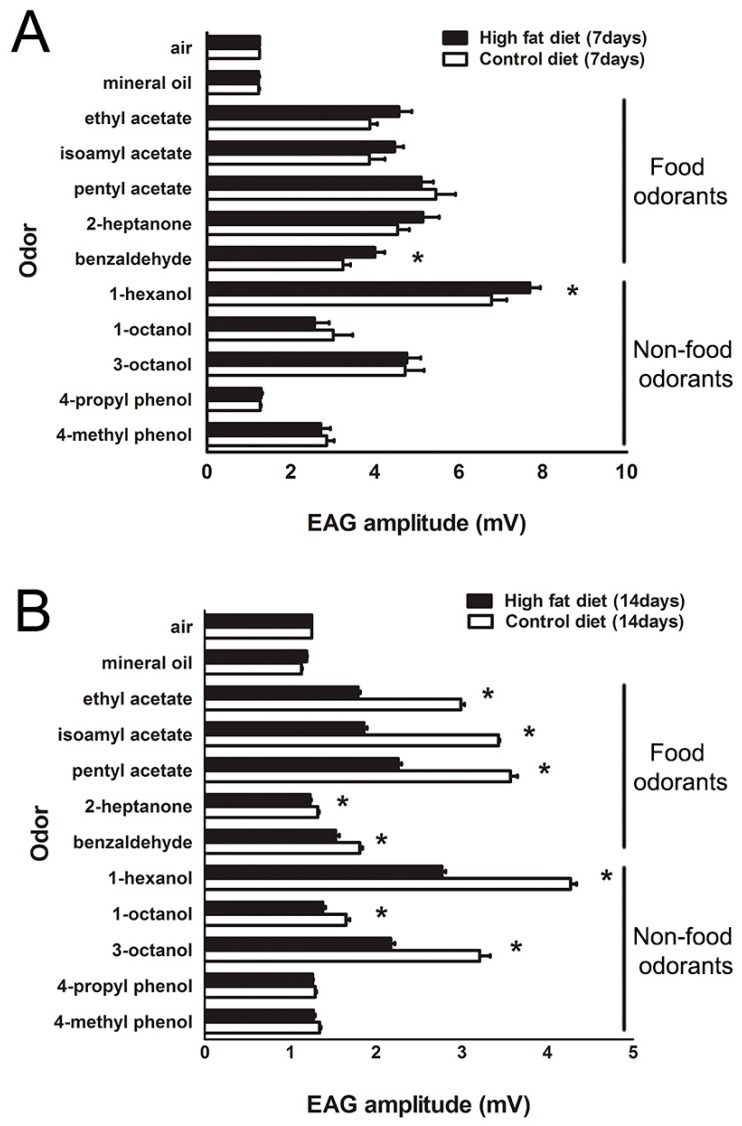
Olfactory responses of wild type Canton-S flies fed with control diet and HFD for seven days and 14 days. (**A**) Flies fed with HFD for seven days showed a reduction of olfactory responses to several odors. (*n* = 15 flies, * *p* < 0.05). Error bars represent SEM. (**B**) Diet treatment for 14 days demonstrated that olfactory sensitivity was mostly decreased to most odorants. Flies fed with HFD for 14 days showed a decline of olfactory responses to several odors, such as ethyl acetate, isoamyl acetate, pentyl acetate, 1-hexanol, and 3-octanol. (*n* = 15 flies, * *p* < 0.05). Error bars represent SEM.

Olfactory sensitivity is known to be affected by internal and external factors [25]. Therefore, we asked whether the HFD affects olfactory sensitivity, including attraction to food odors, such as ethyl acetate, isoamyl acetate, pentyl acetate, 2-heptanone, and benzaldehyde when compared to non-food odors such as 1-hexanol, 3-octanol, 1-octanol, 4-methyl phenol, and 4-propyl phenol. A significant difference in EAG responses was found in flies fed with HFD for seven or 14 days (Figure 2A,B). Flies treated with HFD for 14 days showed decreases in olfactory sensitivities for the food odorants and three of five non-food odorants (Figure 2B); however, the responses of acetate odorants on HFD treatment for seven days had no significant differences between flies on a HFD and standard diet (Figure 2A). It was noting that olfactory sensitivity to benzaldehyde and 1-hexanol increased substantially in flies fed with HFD for seven days (Figure 2A).

### 2.3. Reduction of DmOrco Gene Expression in the Fly Antenna after HFD Treatment

**Figure 3:**
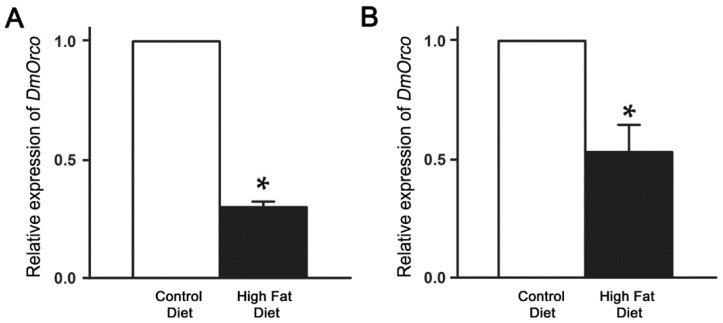
Relative expression of *DmOrco* in antenna of wild type Canton-S flies fed control diet and HFD for seven days and 14 days. (**A**) Relative *DmOrco* mRNA transcript levels after different food treatment for seven days. The level of *DmOrco* transcript was significantly reduced in HFD-fed flies 7 days after food treatment, showing an approximately 70% decrease compared with control flies. (**B**) Relative *DmOrco* mRNA transcript levels after different food treatment for 14 days. All qRT-PCRs were carried out in triplicate (* *p* < 0.05). Error bars represent SEM.

We next asked whether the odorant receptor co-receptor gene of *D. melanogaster* (*DmOrco*), expressed in most olfactory receptor neurons (ORNs) [25], is affected in flies by HFD treatment. The results showed that the level of *DmOrco* transcripts was significantly reduced by approximately 70% in flies fed with HFD for seven days when compared with those of control flies, while it was about a 47% reduction at 14 days (Figure 3A,B).

### 2.4. Modification of Odorant Choice Behaviors by HFD Treatment

**Figure 4:**
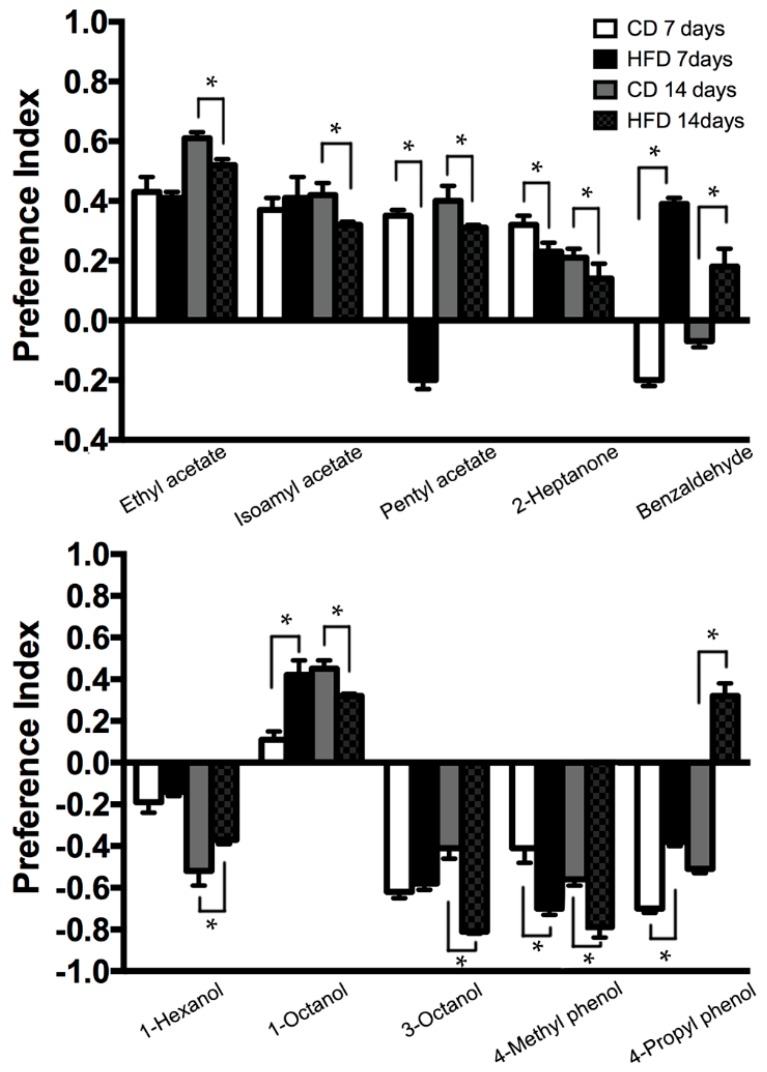
Alteration of attraction behavioral responses of wild type Canton-S flies fed with control diet and HFD for seven days and 14 days to several odors in a T-maze. Flies fed with HFD showed different responses in terms of attraction and repulsion to odorants compared with those fed with the control diet. 20 male flies were tested in each trial. The numbers of flies trapped in the control trap were indicated on the right side of the T-maze, while those in the test trap were indicated on the left side. * *p* < 0.05.

HFD-fed flies showed different responses in terms of attraction and repulsion to the experimental odors as compared with those on the control diet. First, flies on a HFD at 14 days showed a significant decrease in appetitive behavior to ethyl acetate, isoamyl acetate, pentyl acetate, and 2-heptanone. On the other hand, there was an increase in repulsive behavioral responses to 3-octenol (14 days), 4-methyl phenol (7 and 14 days) in flies fed with HFD (Figure 4). In addition, the behavioral responses of HFD-fed flies to benzaldehyde and pentyl acetate showed a transition between repulsive and attractive behavior. In detail, there was an attractive behavioral response to benzaldehyde by flies fed with HFD for seven and 14 days, while the response to pentyl acetate disappeared with HFD for seven days (Figure 4). We found that the behavior of HFD-fed flies to 4-propyl phenol shift from repulsive response to appetitive response (Figure 4). In addition, while HFD-fed flies at seven days exhibit increased attractive response to 1-octanol, flies exposed to HFD at seven days show decreased attractive response to 1-octanol when compared to the response of control diet-fed files (Figure 4). Taken together, the exposure to high levels of dietary fat affects fruit fly olfactory related behavior.

### 2.5. Alteration of Gene Expression Profiles by HFD Treatment

While considering the modification in patterns of olfactory sensitivity and behavior to odorants, we subsequently examined the gene expression levels in the antenna from flies fed with HFD for 14 days. Our Illumina paired-end sequencing yielded a total of 13,287,258 and 11,103,566 raw reads with the length of 101 bp from antenna tissue in flies fed with control diet (CD) and high fat diet (HFD), respectively. Firstly, we quantified our sequencing data by mapping against files transcript sequences. This analysis revealed that 72% of reads were mapped in the control diet-treated flies, while 67% were aligned in HFD-treated flies. Collectively, over two-thirds of the *Drosophila melanogaster* transcriptome data was expressed in both the control and HFD-treated flies, which is a similar observation for gene expression in other insects [26].

**Figure 5:**
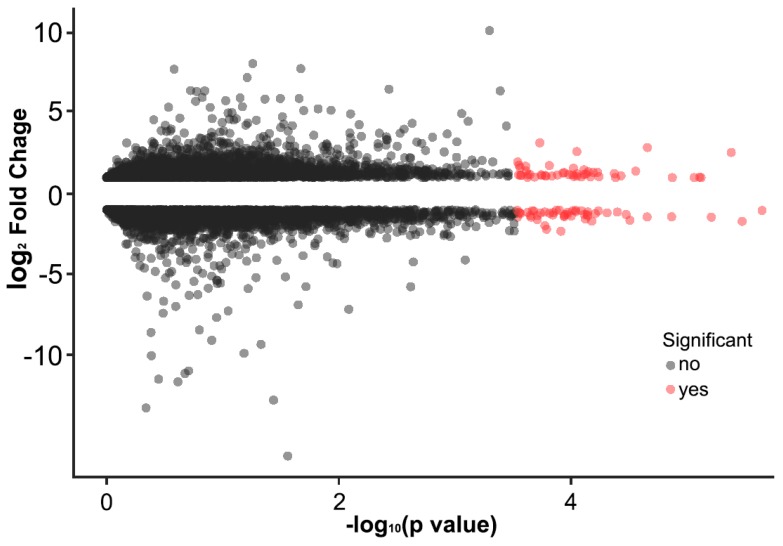
Gene expression changes in antennae of files on high fat diet. The x-axis is the −log10 *p*-value and the y axis is the fold-change value. Using the *p*-value 0.05 as the threshold cutoff, 97 genes in the left and right are selected. Red spots indicate the statistically significant differentially expressed genes (DEGs).

Then, we attempted to identify differentially expressed genes between control diet and HFD. A significant change in the level of gene expression after HFD treatment was indicated for a total of 732 genes, of which 379 genes were up-regulated and 343 genes down-regulated (Figure 5). From functional annotation, we noticeably identified genes that are related to olfactory related genes and neuronal sensor, which were down-regulated (Table 1). A total of 74 olfactory genes, including 34 odorant receptors (ORs) and 30 odorant-binding proteins (OBPs) were up-regulated and down-regulated from antennae of flies on high fat diet (Table 2 and Table 3).

**Figure 6:**
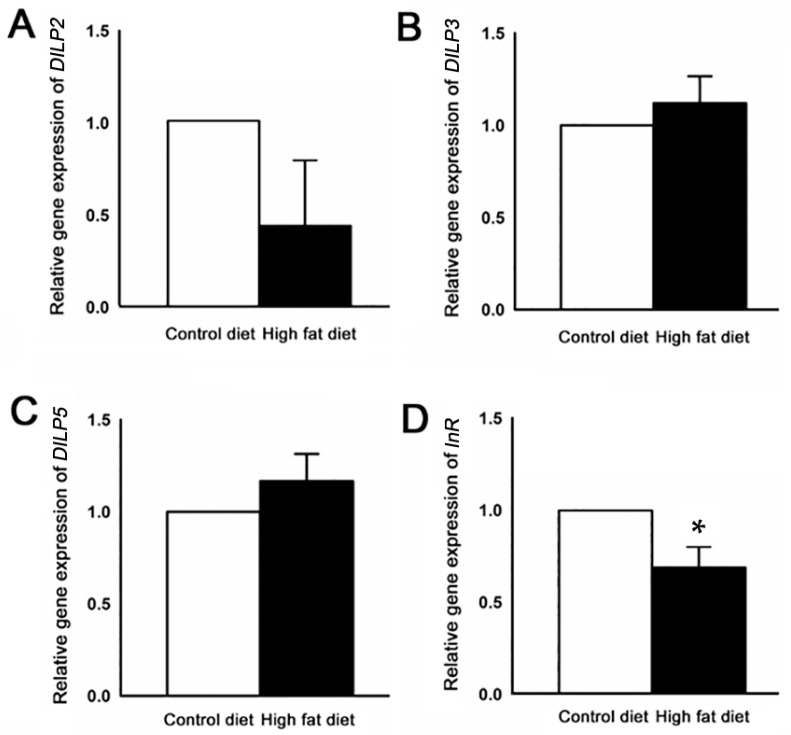
Gene expression levels of some target genes related to insulin signaling identified by DEG analysis. (**A**) Relative *DILP2* mRNA transcript levels after different food treatments for 14 days. (**B**) Relative *DILP3* and (**C**) *DILP5* mRNA transcript levels after different food treatments for 14 days. Both of the gene expressions were increased by approximately 10% in high fat diet (HFD)-fed flies as compared with control flies. (**D**) Relative *InR* mRNA transcript levels after different food treatments for 14 days. *InR* gene expression level was significantly decreased by approximately 30% compared with control flies. All qRT-PCRs were carried out in triplicate (* *p* < 0.05). Error bars represent SEM.

Next, we analyzed the expression of the genes associated with insulin signaling pathways (Table S1). Normalization of qRT-PCR was performed for four reference genes that are involved with insulin signaling genes to increase accuracy. We measured the transcript variation of *DILP2*, *DILP3*, *DILP5*, and *InR* genes for data normalization. Although the relative levels of *DILP2*, *DILP3*, and *DILP5* expression showed no significant differences, *InR* expression demonstrated a significant decrease (Figure 6).

**Figure 7:**
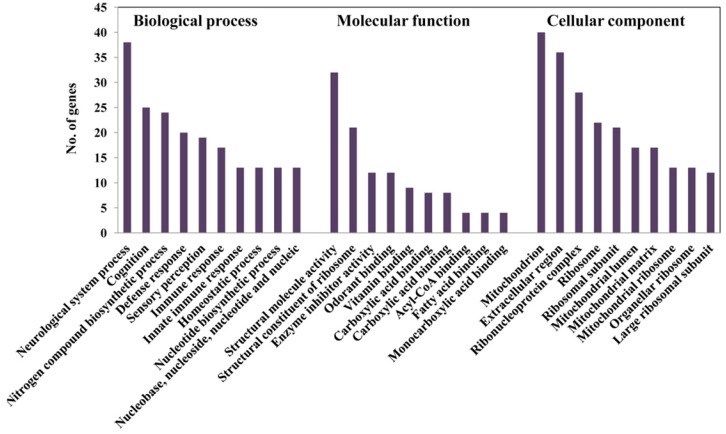
Gene Ontology (GO) analysis of expressed genes showing log_2_ fold changes between control diet (CD) and high fat diet (HFD). GO terms in the biological process, molecular function, and cellular component were shown.

To find out the main functional process that is affected by HFD treatment, we categorized the genes whose expression exhibited the substantial changes after HFD treatment. We analyzed the genes with a more than log two-fold change in expression in up- and down-regulation. These data were established according to the functional groups of GO, including biological process (BP), molecular function (MF), and cellular component (CC) (Figure 7). In particular, the largest number of genes was representative of several categories of BP, namely neurological system process, cognition, and sensory perception (Figure 7), which suggests a relevance to olfactory responses in both the peripheral and central nervous systems. Furthermore, genes of defense response and immune response were associated with response to HFD treatment.

## 3. Discussion

### A High-Fat Diet Leads to Olfactory Dysfunction in Homeostatic Processing in Drosophila

High fat diet (HFD) is associated with an increased risk for disease, including obesity, heart disease, diabetes, and cancer [5,6,7]. In the present study, HFD for 14 days lead to a loss of climbing ability and reduction of life span of flies. Although consumption of HFD are found to cause nervous system dysfunction, whether such diets affect peripheral sensory system is largely unknown. We discovered a relationship between HFD and olfactory dysfunction in the fruit fly. Excessive stress by a HFD has a crucial impact on olfactory sensitivity. HFD-treated flies for 14 days were less sensitive to five food odors and three non-food odors. This is consistent with a recent reported that mice on HFD exhibited decreased olfactory discrimination [27]. This reduction in sensory sensitivity, which is exposed by a chronic fat diet, may be linked to the significantly decrease of olfactory related genes or other neuromodulators. For example, our DEG analysis indicated that 27 olfactory receptor genes and 21 odorant binding protein genes were down regulated in flies on fat diet. Because ORs and OBPs have been demonstrated to play an important role in olfactory receptor neurons maturation and axon guidance [28], one speculation is that high fat diet induced the down regulation of OR and OBP expression in olfactory receptor neuron that might thereby interfere with appropriate gene processing and targeting olfactory receptor neuronal axons to the proper antennal lobe region [29]. The cellular mechanisms underlying the HFD induced regulation of olfactory receptor gene expression are largely unknown. Recent evidence has emerged that proinflammatory responses are evoked in the olfactory epithelium in response to altered energy consumption [30]. HFD induce an increase in the number of macrophages and neuronal death, which resulted in a loss of connections from the olfactory epithelium and olfactory bulb [31]. Our DEG data showed that there was an upregulation of two genes that are known to be involved in apoptotic processes: *Traf6* and *Decay*. Increased expression of these genes may induce apoptotic death in response to HFD. Further experiments are necessary to demonstrate whether *Traf6* and *Decay* can induce apoptotic death of olfactory receptor neurons. Our DEG analysis provides the possibility that some mediators may regulate expression of the olfactory receptor genes and odorant binding proteins. Certain genes were observed in functional changes, which is associated with olfactory and nutrient-related pathways that modulate olfactory responses in flies. Previous studies demonstrated microarray analysis after starvation [32] or heat treatment [26] that affected olfactory responses and feeding behaviors, depending on nutritional status in adult flies. In both cases, the analysis included the antennal part, which is the main olfactory receptor organ, to make clear the importance of the internal state of olfaction.

One of the most prominent changes we found was a reduced appetitive behavioral response to olfactory stimuli, suggesting that HFD feeding may cause the alteration in olfactory perception. This is consistent with a recent report that mice with HFD-induced obesity showed deficits in olfactory learning and memory formation and altered behaviors related to olfaction and taste [33]. Besides, obese people have been shown to prefer a food-related odorant rather than a non-food related odorant when compared with normal weight people as same ages, implying that these people might not be able to regulate food intake by their internal state through appropriate odor processing [8]. Although molecular and neural mechanisms underlying the HFD-induced modification of olfactory behavior are not addressed yet, many lines of the research have been focused on insulin resistance. It has been reported that the activation of food stress responses is involved in the induction of insulin resistance and interestingly these results are observed in the olfactory bulb [34], implying that food stress-induced insulin resistance may affect the sensory cognition ability in animals.

In *Drosophila*, local signals by the neuropeptides and global metabolic cues by insulin are modified at specific olfactory sensory neuron to change olfactory responses [21,35,36]. For example, insulin as a metabolic factor modulates olfactory responses in fly antennae by enhancing the pre-synaptic facilitation and regulating odor preference in accordance with the internal state [18]. Furthermore, *Drosophila* insulin-like peptide 2 (DILP2) was shown to modulate a short neuropeptide F receptor (sNPFR1), which regulates olfactory driven behaviors in antenna and is also stimulated in starved flies [37,38,39]. Similarly, several studies demonstrate the importance of neuropeptides in the regulation of olfactory behavior [40,41]. Our digital gene expression analysis detected transcripts of *DILP2* and insulin receptor (*InR*) in significant changes of down-regulation, in contrast with other insulin-related genes. This could be partially explained by the reduction of olfactory sensitivity caused by HFD treatment, and this may cause a loss of controlling ability of attractive behaviors to food odors. Recent research has demonstrated that *DILP2* shows different expression levels depending on the type of diet [42]. Therefore, high fat diet condition must be function with other peripheral receptors, including those for olfaction. Olfactory responses to HFD treatment are affected by the over-expression of certain insulin signaling genes; therefore, communication through olfactory modulation could contribute to peripheral organs and subsequently be linked to the central nervous system [13,43].

Interestingly, the behavioral responses of HFD-fed flies to benzaldehyde and pentyl acetate showed a transition between repulsive and attractive behavior, which is contrary to the expected physiological responses of the fly. This defective olfactory behavior might be associated with the misexpression of specific genes. Our DEG analysis showed that transcript levels of several olfactory receptor genes and odorant binding protein genes were changed. Among these genes, misexpression of the *or43a*, one of the benzaldehyde receptors, caused a reduction of behavioral avoidance responses to benzaldehyde [44]. In addition, *lush* is a soluble odorant binding protein and it is expressed exclusively in the chemosensory system in flies. Mutants of this gene have been reported to a loss of the avoidance behaviors [45] and defects for pheromone-evoked behaviors [46]. Since the genetic dissection by which genes modulate olfactory behavior about each odorant remains unclear, further genetic experiments are necessary to demonstrate the relevance of specific olfactory sensory input and subsequent processing for olfactory driven behaviors.

## 4. Materials and Methods

### 4.1. Drosophila Stocks and Diet Treatment

The Canton-S wild-type strain of *Drosophila melanogaster* that was obtained from Bloomington Stock Center (Bloomington, IN, USA) was used in this study. All of the adult flies were maintained at 25 °C with 60% relative humidity and a 12 h light/12 h dark cycle. Food media for a standard diet was prepared by mixing 10% sugar, 10% yeast extract, and 1.5% agar with 1% Tween 80 (*w*/*v*) added in case of toxicity. To make the high-fat diet, 2% palmitic acid (*w*/*v*) and 1% Tween 80 (*w*/*v*) were added into the standard diet in accordance with a previous study [22].

In order to obtain adult flies for the experiments, newly enclosed flies were collected into new bottles and kept for two days on the standard diet. Next day, male and female flies were sorted under CO_2_ anesthesia and only male flies were placed on the standard diet for one more day. Test male flies were treated with the standard diet and a high-fat diet (HFD) for seven days and 14 days, respectively. Each vial contained 20 male flies.

### 4.2. Life Span and Climbing Assays

For life span assays, flies were kept at a density of 20 male flies per each vial on the standard diet and HFD. Each group of flies was transferred to a vial with fresh food medium every three days and dead flies in each group were counted every day. Five replicates were conducted for this experiment.

For climbing assays, a behavioral paradigm that was reported in a previous study was employed with slight modifications [24]. Twenty male flies were transferred to a 10 cm glass vial and placed at the bottom of the vial by gently tapping the flies down to the bottom. The number of flies that climbed above 80% height within 30 s was counted. Experimental trials were repeated ten times independently for each group. The climbing index (%) was calculated as the ratio of the number of flies climbing above 80% height to the total number of flies multiplied by 100. All of the climbing assay experiments were performed at 25 °C with 60% humidity.

### 4.3. Odor Stimulation

All the odorants tested in this study (ethyl acetate, isoamyl acetate, pentyl acetate, benzaldehyde, 2-heptanone, 1-hexanol, 3-octanol, 1-octanol, 4-propyl phenol, and 4-methyl phenol) were commercially available products at the highest purity (>98%, Sigma-Aldrich, Milwaukee, WI, USA). These odorants were dissolved in mineral oil (Sigma-Aldrich-330760) or ethanol at 1% dilution (*v*/*v*). A glass tube was prepared with a continuous, humidified air stream, and 20 μL of odorant solution was soaked onto a filter paper (4 × 4 mm, Toyo Roshi Kaisha, Tokyo, Japan) that was placed into a 5 mL disposable syringe with air pressure. These chemicals were used as odorant sources for EAG recordings and for behavioral assays.

### 4.4. Electrophysiological Recordings

Transepithelial electrophysiological recordings (electroantennograms, EAGs) from antennae in male Canton-S flies were made after individual diet treatment while using AgCl-coated silver wire inserted in a glass micropipette filled with 0.1 M KCl. The experimental fly was immobilized in a truncated 200 μL plastic pipette tip and trimmed to show an anterior aspect of the fly’s head. The third segments of the antennae were exposed for EAG recordings. A reference electrode was inserted into a compound eye and a recording electrode was placed on the dorso-medial surface of the third antennal segment, as described previously [47]. Electrical signals were amplified with an analog 10× active probe and conveyed to an acquisition system (IDAC4, Syntech, Hilversum, The Netherlands). Signals were then further recorded and analyzed while using EAG Pro software (Syntech). A constant air stream of 40 mL/min was delivered to the fly head by using a stimulus controller (CS-55, Syntech). Pulses of the odorants were produced during a 1 s stimulation through the Pasteur pipette to the syringe pipette. Signal amplitude of olfactory responses (mV) was measured from a baseline before stimulation to the trough of the electrical signals after odorant stimulation. Control EAG experiments loaded with no odorant and mineral oil were used. The interval between each odorant stimulus was about 60 s to prevent the adaptation of the test animal to a given odorant.

### 4.5. Quantitative RT-PCR

Flies were collected according to the procedures that are presented above for the diet treatment. Total RNA extraction was carried out while using an RNeasy Mini Kit (Qiagen, Valencia, CA, USA), according to the manufacturer’s protocol. For each treatment, at least three independent extractions were conducted while using 100 fly antennae. The quality and quantity of total RNA was measured by a Nanodrop 2000 (Thermo Scientific Inc., Wilmington, DE, USA). cDNA was prepared from the extracted RNA while using the Superscript III First-Strand Synthesis kit (Invitrogen Inc., Carlsbad, CA, USA). Quantitative RT-PCR was performed using a StepOnePlus machine (Applied Biosystems Inc., Foster City, CA, USA) according to the manufacturer’s protocol with SYBR green qRT-PCR Master Mix (Fermentas, Burlington, ON, Canada) to measure *DmOrco* gene expression. qRT-PCR measurement for each gene was repeated with three independent biological samples and quantitative analysis was conducted by StepOne plus Software V. 2.0 (Applied Biosystems). The transcript level of the gene was calculated by the standard curve method and normalized to the control gene *ribosomal protein 49 (rp49)* primer that was described in a previous paper [48]. The primer sequences for *DmOrco* were forward: 5′-GGTGGACCATGAGACGAACT-3′; reverse: 5′-CATCACGTCGCATAGATTGG-3′.

### 4.6. Behavioral Assay

Flies were placed under light CO_2_ anesthesia. Separated male flies were placed into vials (20 flies per vial) containing each diet treatment. Flies were transferred to new vials every two to three days and behavioral assays performed on days 7 and 14 after diet treatment. One day before the assay, flies were moved into empty vials containing only water on Kimwipes (Kimberly-Clark Worldwide, Neenah, WI, USA) for at least 15 h to produce a starved state [49]. For each measurement, the flies were gently tapped to place into a T-maze. The same odorants as used for the EAGs were used for this behavioral assay. Each vial at both sides contained two traps, an odor trap, and a control trap (water). Flies were allowed to have 30 s to choose one of the sides in the T-maze that contained odorant. The attraction index (AI) was calculated, as follows: (number of flies moving toward the odor trap − number of flies in the control trap)/(total number of flies). Ten replicates were performed for each treatment. All T-maze assay experiments were conducted at 25 °C with 60% humidity.

### 4.7. Analysis for Differentially Expressed Genes (DEG) and Gene Ontology of HFD-Fed Flies

Approximately 3000 samples of third antennal segments of male adult flies were prepared to achieve a sufficient representation of genes. The flies in each diet treatment were collected from 11:00 to 14:00 and 16:00 to 19:00 to prevent the possible modification of gene expression by circadian rhythms [26]. All the samples were incorporated to minimize any random factors that affect gene expression other than the treatment. Gene sequences were downloaded from the Drosophila Genome database (www.flybase.org) for the analysis of differentially expressed genes (DEG).

For the RNA sequencing experiment, 4 μg of total RNA was extracted while using the same protocol, as described above in the quantitative RT-PCR procedure. In order to convert mRNA in total RNA into a library of template molecules for subsequent cluster generation, a Illumina^®^ TruSeq™ RNA sample preparation kit was used in accordance with the manufacturer’s instructions (Illumina Inc., San Diego, CA, USA). The total RNA sample was used for poly-A mRNA selection using poly-T oligo-attached magnetic beads with two rounds of purification. The resulting mRNA sample from the antennae was subjected to thermal mRNA fragmentation while using Elute, Prime, Fragment Mix from the Illumina^®^ TruSeq™ RNA sample preparation kit. The mRNA fragments were reverse transcribed to synthesize the first-strand cDNA using a combination of reverse transcriptase and random primers. The mRNA template strand was removed and double-stranded cDNA (ds cDNA) was generated using DNA polymerase І. The ds cDNA was purified using Ampure XP beads to separate the ds cDNA from the second-strand reaction mix.

The cDNA fragments were then blunt-ended through an end-repair reaction while using an End Repair (ERP) mix (Illumina Inc). The 3′ to 5′ exonuclease activity of this mix removed the 3′ overhangs and the polymerase activity filled in the 5′ overhangs. Next, “A” nucleotide was added to the 3′ ends of the blunt fragments to prevent them from ligating to each other and then ligated to platform-specific double-stranded bar-coded adapters that provided a complementary overhang during the adapter ligation reaction. DNA libraries were sequenced using an Illumina HiSeq2000. The sequencing reads were deposited to the NCBI SRA (Short Read Archive) site (https://www.ncbi.nlm.nih.gov/sra) under the accession number SRR1501071 for the control diet and SRR1501074 for the high fat diet.

DEG analysis was performed using edgeR bioconductor package and RSEM software [50]. Quality check, normalization, and statistical analysis were performed to develop significantly matched signals. Genes with false discovery rate (FDR) value of at most 0.001 and fold change value of ≥2 were considered as significant differentially expressed genes. In addition, log_2_ (FPKM) expressional difference was manually calculated for all of the genes based on FPKM (Fragments Per Kilobase Million) values. After obtaining general information on the gene expression, gene ontology (GO) analysis was carried out for genes that showed log_2_ fold digital gene expression difference after diet treatment using the DAVID tool (http://david.abcc.ncifcrf.gov/). In the DAVID annotation system, modified Fisher Exact *p* value (EASE score) was adopted to measure the gene-enrichment in annotation terms. All data analysis and visualization of differentially expressed genes were conducted while using R 2.15.1 (http://www.r-project.org).

### 4.8. Statistical Analysis

Statistical analyses were performed by Student’s t-test (SPSS, Version 20, IBM, New York, NY, USA) on the results from the lifespan assay, climbing ability test, EAGs, and T-maze tests to determine any significant differences between the control diet and HFD treatment. Data were shown as means ± standard error.

## 5. Conclusions

High-fat diet affects sensory abilities in *Drosophila melanogaster* such as olfactory-driven behaviors and olfactory sensitivity to several odorants, which in turn makes some changes in molecular levels of gene expressions. Integration of internal and external factors provides crucial evidence to olfactory modulation in animals. Our present study demonstrates that high fat diet alters olfactory perception and changes of gene expression profiles in *Drosophila melanogaster*. A better understanding of the complex mechanisms that are underlying olfactory modulation might help to characterize the olfactory systems that are affected by HFD treatment. Identifying novel regulators of the olfactory system and examining them to find out specific odorant receptors to odors at the intracellular level affected by HFD treatment may provide an understanding of the mechanisms and circuits from antenna to brain in flies.

## Figures and Tables

**Table 1:** Functional description for differentially expressed genes.

Gene ID	Functional Description	Gene ID	Functional Description
**Upregulated**		**Downregulated**	
FBtr0086664	IM2	FBtr0025595	Akhr
FBtr0028381	Decay	FBgn0052523	Serine protease
FBtr0070099	Cyp4g1	FBgn0026389	Or43a
FBtr0100231	RpL41	FBtr0082607	GstD1
FBtr0086662	IM1	FBtr0073062	Drsl5
FBtr0074176	sun	FBgn0053532	Lectin-37Da
FBtr0088035	eEF1α1	FBgn0037324	Orco
FBtr0073468	antdh	FBtr0085805	RpL6
FBtr0087992	Cyp6g1	FBgn0036926	CG7646
FBgn0001179	Hay	FBgn0026385	Or47b
FBtr0072924	RpL8	FBtr0074969	lush
FBtr0307212	CG16978	FBtr0079455	Obp28a
FBtr0086477	Obp56d	FBtr0076229	Sod1
FBtr0072185	RpL39	FBgn0036009	Or67a
FBgn0013343	Syntaxin-1A	FBtr0081427	CG9336
FBgn0035505	Teh2	FBtr0081855	COX7A
FBtr0113742	RpL15	FBtr0086292	Obp57c
FBtr0086216	CG18067	FBtr0087494	CG30197
FBtr0081597	Obp84a	FBgn0038798	Or92a
FBgn0033483	Egr	FBgn0034474	Obp56g
FBtr0080306	CG6770	FBtr0071135	RpS6
FBtr0265464	Traf6	FBtr0078769	RpL35A
FBtr0084410	RpS3	FBgn0034475	Obp56h
FBtr0300322	Sfp84E	FBgn0033789	CG13324
FBtr0085463	Obp99c	FBgn0038203	Or88a
FBgn0053502	CG33502	FBtr0088525	RpL31
FBtr0070983	RpL17	FBtr0082370	RpS25
FBtr0087747	CG4716	FBtr0086672	GstE4
FBgn0041625	Or65a	FBgn0028416	Met75Ca
FBgn0036078	Or67c	FBtr0071935	RpS24
FBtr0071096	RpS14b	FBtr0075217	CG7630
FBtr0075884	RpS4	FBgn0032008	CG14277
FBtr0300635	CG42502	FBtr0070290	sta
FBtr0087105	RpLP2	FBtr0307166	Obp19a
FBtr0072805	RpL23A	FBtr0302899	CG6503
FBtr0305965	CG14661	FBtr0073097	RpL28
FBtr0075839	Nplp2	FBtr0078656	Obp83a
FBtr0075955	Obp69a	FBtr0072164	EbpIII
FBtr0075066	RpL26	FBtr0112740	ND-MWFE
FBtr0077470	RpL40	FBtr0071392	CG9691
FBtr0076423	RpS9	FBtr0307366	lncRNA:CR34335
FBtr0082962	His4r	FBtr040734	CG15065
FBtr0081920	CG8369	FBtr0077922	a5
FBtr0072173	eEF5	FBtr0040735	CG16386
FBtr0071360	RpS28b	FBtr0083969	RpS30
FBtr0076273	CG6409	FBtr0075290	a10
FBtr0078655	Obp83b	FBtr0300828	RpS15Aa
FBtr0300321	Os-C	FBtr0076032	RpL10Ab
		FBtr0332183	OS9

**Table 2:** Olfactory receptor (Or) genes: Down- and up-regulated after 14 days HFD treatment.

Gene ID	Functional Description	Gene ID	Functional Description
**Upregulated**		**Downregulated**	
FBgn0026398	Or22a	FBgn0037324	Orco
FBgn0026397	Or22b	FBgn0030204	Or9a
FBgn0041625	Or65a	FBgn0041626	Or19a
FBgn0036078	Or67c	FBgn0062565	Or19b
FBgn0037399	Or83c	FBgn0026395	Or23a
FBgn0037685	Or85f	FBgn0026392	Or33a
		FBgn0026391	Or33b
		FBgn0028946	Or35a
		FBgn0033041	Or42a
		FBgn0033043	Or42b
		FBgn0026389	Or43a
		FBgn0033404	Or45a
		FBgn0026386	Or47a
		FBgn0026385	Or47b
		FBgn0028963	Or49b
		FBgn0034473	Or56a
		FBgn0041624	Or65b
		FBgn0041623	Or65c
		FBgn0036009	Or67a
		FBgn0036019	Or67b
		FBgn0041622	Or69a
		FBgn0037576	Or85a
		FBgn0026399	Or85e
		FBgn0038203	Or88a
		FBgn0038798	Or92a
		FBgn0039551	Or98a
		FBgn0038798	Or92a

**Table 3:** Odorant-binding protein (Obp) genes: Down- and up-regulated after 14 days HFD treatment.

Gene ID	Functional Description	Gene ID	Functional description
**Upregulated**		**Downregulated**	
FBgn0050067	Obp50a	FBgn0030103	Obp8a
FBgn0033931	Obp50e	FBgn0030985	Obp18a
FBgn0034468	Obp56a	FBgn0031109	Obp19a
FBgn0034470	Obp56d	FBgn0031110	Obp19b
FBtr0075955	Obp69a	FBgn0031111	Obp19c
FBgn0046876	Obp83ef	FBgn0050052	Obp49a
FBtr0078655	Obp83b	FBgn0043530	Obp51a
FBtr0081597	Obp84a	FBgn0046879	Obp56c
FBgn0039682	Obp99c	FBgn0034471	Obp56e
		FBgn0043533	Obp56f
		FBgn0034474	Obp56g
		FBgn0034475	Obp56h
		FBgn0034509	Obp57c
		FBgn0050145	Obp57e
		FBgn0034768	Obp58b
		FBgn0034769	Obp58c
		FBgn0034770	Obp58d
		FBgn0034766	Obp59a
		FBgn0046875	Obp83a
		FBgn0039685	Obp99b
		FBgn0039684	Obp99d

## Supplementary Materials

Supplementary materials can be found at http://www.mdpi.com/1422-0067/19/10/2855/s1.
