# Supplementary material for: The Effects of High Fat Diet-Induced Stress on Olfactory Sensitivity, Behaviors, and Transcriptional Profiling in Drosophila melanogaster
Source: Int J Mol Sci. 2018 Sep 20;19(10):2855. doi: 10.3390/ijms19102855 (PMC6213603; doi:10.3390/ijms19102855)
Supplement: Supplementary file 1 [file ijms-19-02855-s001.pdf]

Supplementary Table 1. Expression profiling of candidate genes involved in insulin pathway.

Gene symbol	Flybase Idx	Expression level based on FPKM (High fat diet)	Expression level based on FPKM (Control diet)	Fold-change
**DILP2**	FBgn0036046	9.76	50.32	-2.36
**DILP3**	FBgn0044050	12.34	9.32	0.40
**DILP6**	FBgn0044047	129.63	32.76	1.98
**DILP7**	FBgn0044046	2.73	9.37	-1.77
**DILP8**	FBgn0036690	17.88	12.76	0.49
**Chico**	FBgn0024248	173.48	230.87	-0.41
**InR**	FBgn0013984	283.45	784.82	-1.47
**Pten**	FBgn0026379	78.32	140.87	-0.84
